# Biomarkers of Airway Disease, Barrett’s and Underdiagnosed Reflux Noninvasively (BAD-BURN): a Case-Control Observational Study Protocol

**DOI:** 10.21203/rs.3.rs-4355584/v1

**Published:** 2024-05-15

**Authors:** Urooj Javed, Sanjiti Podury, Sophia Kwon, Mengling Liu, Daniel Kim, Aida Fallah Zadeh, Yiwei Li, Abraham Khan, Fritz Francois, Theresa Schwartz, Rachel Zeig-Owens, Gabrielle Grunig, Arul Veerappan, Joanna Zhou, George Crowley, David Prezant, Anna Nolan

**Affiliations:** New York University Grossman School of Medicine (NYUGSoM); New York University Grossman School of Medicine (NYUGSoM); New York University Grossman School of Medicine (NYUGSoM); New York University Grossman School of Medicine (NYUGSoM); New York University Grossman School of Medicine (NYUGSoM); New York University Grossman School of Medicine (NYUGSoM); New York University Grossman School of Medicine (NYUGSoM); New York University Grossman School of Medicine (NYUGSoM); New York University Grossman School of Medicine (NYUGSoM); New York University Grossman School of Medicine (NYUGSoM); New York University Grossman School of Medicine (NYUGSoM); New York University Grossman School of Medicine (NYUGSoM); New York University Grossman School of Medicine (NYUGSoM); New York University Grossman School of Medicine (NYUGSoM); New York University Grossman School of Medicine (NYUGSoM); New York University Grossman School of Medicine (NYUGSoM); New York University Grossman School of Medicine (NYUGSoM)

**Keywords:** Air Pollutants, Airway hyperreactivity, Ambient Particulate Matter, Barrett’s Esophagus, Gastro-Esophageal Refux Disease, Particulate, Aerodigestive

## Abstract

**BACKGROUND.:**

Particulate matter exposure (PM) is a cause of aerodigestive disease globally. The destruction of the World Trade Center (WTC) exposed fifirst responders and inhabitants of New York City to WTC-PM and caused obstructive airways disease (OAD), gastroesophageal Refux disease (GERD) and Barrett’s Esophagus (BE). GERD not only diminishes health-related quality of life but also gives rise to complications that extend beyond the scope of BE. GERD can incite or exacerbate allergies, sinusitis, bronchitis, and asthma. Disease features of the aerodigestive axis can overlap, often necessitating more invasive diagnostic testing and treatment modalities. This presents a need to develop novel non-invasive biomarkers of GERD, BE, airway hyperreactivity (AHR), treatment efficacy, and severity of symptoms.

**METHODS.:**

Our observational case-cohort study will leverage the longitudinally phenotyped Fire Department of New York (FDNY)-WTC exposed cohort to identify *Biomarkers of Airway Disease, Barrett’s and Underdiagnosed Refux Noninvasively (BAD-BURN)*. Our study population consists of n = 4,192 individuals from which we have randomly selected a sub-cohort control group (n = 837). We will then recruit subgroups of *i*. AHR only *ii*. GERD only *iii*. BE *iv*. GERD/BE and AHR overlap or *v*. No GERD or AHR, from the sub-cohort control group. We will then phenotype and examine non-invasive biomarkers of these subgroups to identify under-diagnosis and/or treatment efficacy. The findings may further contribute to the development of future biologically plausible therapies, ultimately enhance patient care and quality of life.

**DISCUSSION.:**

Although many studies have suggested interdependence between airway and digestive diseases, the causative factors and specific mechanisms remain unclear. The detection of the disease is further complicated by the invasiveness of conventional GERD diagnosis procedures and the limited availability of disease-specific biomarkers. The management of Refux is important, as it directly increases risk of cancer and negatively impacts quality of life. Therefore, it is vital to develop novel noninvasive disease markers that can effectively phenotype, facilitate early diagnosis of premalignant disease and identify potential therapeutic targets to improve patient care.

**TRIAL REGISTRATION:**

ClinicalTrials.gov Identifier: NCT05216133; January 18, 2022.

## BACKGROUND

Particulate matter (PM) exposure is a risk factor for aerodigestive disease and mortality.^1–3^ On September 11, 2001 (9/11), fifirst-responders and inhabitants of New York City were exposed to World Trade Center (WTC)-PM.^4–35^ Many subsequently developed aerodigestive diseases including obstructive airways disease (OAD), gastroesophageal Refux disease (GERD) and Barrett’s Esophagus (BE).^23,34,36–42^ By 2005, approximately 44% of WTC rescue and recovery workers had developed GERD, which is 8.2-fold higher than the pre-9/11 prevalence, and more than double the general US population.^43–46^ After WTC-PM exposure, GERD occurred more often in asthmatics.^42^ Comorbid aerodigestive disease affected 51.4% of firefighters.^47^

GERD and BE are risk factors for esophageal adenocarcinomas (EAC).^48^ Patients with BE face at least 30-fold higher risk of developing EAC than the general population.^49,50^ Complications of GERD extend beyond malignancy and can adversely affect quality of life (QoL), impair productivity, and lifespan.^46,51–53^ GERD can incite or exacerbate co-morbidities such as allergies, sinusitis, chronic bronchitis, and asthma.^54^ There is a 59.2% prevalence of GERD symptoms in patients with asthma compared to 38.1% in controls.^55^ GERD treatment in WTC responders with proton pump inhibitors (PPIs) have been found to increase risk of severe cognitive impairment.^56^ Cognitive decline with PPI use has also been reported in the general population.^57^

Despite numerous studies suggesting potential interdependence between airway and digestive diseases, the underlying causative factors and mechanisms remain unclear.^55^ Biomarkers are often key to identifying causative pathways and mechanistic targets. While some studies have investigated serum, salivary, and microbial biomarkers of GERD, they are often not focused on the contribution of respiratory disease.^58–60^

The availability of clinical longitudinal phenotyping makes the WTC-PM exposed Fire Department of New York (FDNY) first responders cohort ideal for biomarker discovery.^10,22,28–31,61–65^ Notably, we have successfully identified biomarkers associated with GERD and BE in a pilot population with respiratory disease, facilitating the identification of biologically relevant immune pathways.^3^

The diagnosis of GERD itself is a complex process that relies on subjective clinical symptoms and often necessitate objective but invasive testing such as endoscopy and 24-hour pH monitoring.^66^ Those with endoscopic evidence of Refux may be entirely asymptomatic, potentially leading to under-diagnosis of patients at risk of BE and EAC.^67,68^ Even with the most invasive procedures, the diagnosis of GERD can be elusive and plagued by poor sensitivity.^69^

**Figure 1.**
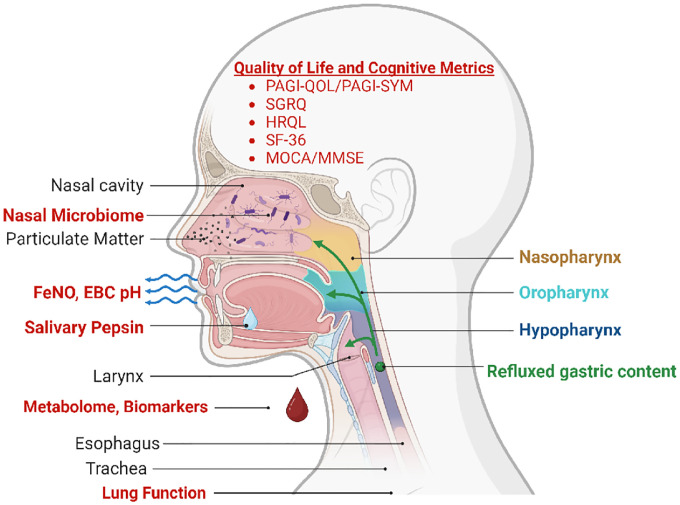
Overview of Planned Biomarker Assessments. **Planned assessments** of reflux **(green)** of gastric material frorm esophagus to nearby trachea, oral cavity, and nasopharynx **(red)** will include, microbiome (nasopharyngeal reflux), fraction of exhaled nitric oxide (FeNO), exhaled breath condensate (histamine and pH), oral cavity (salivary pepsin), and serum (metabolome and biomarkers). In addition, we will assess: Quality-of-life, Reflux related, cognition anc respiratory metrics. Aliquots of all specimens will be banked. Created with BioRender.com.

In light of this, we propose to explore noninvasive biomarkers that could identify a population of aerodigestive disease, enabling better phenotyping of FDNY-WTC cohort with aerodigestive disease. In addition to their diagnostic utility, noninvasive biomarkers may direct future research into mechanisms and their downstream effects. GERD/BE biomarkers are also important to identify in the clinically silent presentations.^69^ Additionally, we will identify novel non-invasive biomarkers of aerodigestive disease through a multi-OMIC approach. We will profile not only the metabolome and microbiome, but also exhaled, secreted, and blood biomarkers of aerodigestive disease, Fig. 1.^70^

To address a critical gap in the current literature, we will **1. Quantify noninvasive measures** of aerodigestive disease (salivary pepsin, serum biomarkers/metabolome, fractional exhaled nitric oxide (FeNO), exhaled breath condensate (EBC), microbiome, cognitive measures and aerodigestive QoL/disease severity measures to phenotype and assess treatment efficacy. **2. Develop and optimize** a noninvasive biomarker model of aerodigestive disease and also **3**. determine the effect of aerodigestive disease on QoL, cognition and symptom phenotype.

## METHODS/DESIGN

### Study Design and Participants.

The FDNY WTC-health program (WTC-HP) electronic medical record (EMR) will be used to obtain clinical variables such as age, gender, years of FDNY service, WTC site exposure level, and lung function measures, as previously described.^22,27,62–65,71^ Our observational study is NYU IRB Approved # 21–00679 and available at clinicaltrials.gov #NCT05216133. Study Definitions and Inclusion/Exclusion Criteria can be found in Table 1.

### Study Population:

**Figure 2.**
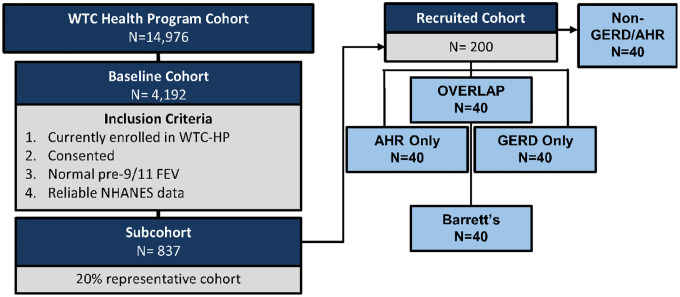
Study Design. Subjects will be recruited for the randomly selected subcohort from population from each of the following groups i. AHR, ii. GERD, iii. Barrett’s, iv. overlap and v. non-GERD/AHR populations.

*Source Cohort*. All participants in the WTC-HP (n = 14,976) were screened, Fig. 2. *Inclusion Criteria*: i. Actively consented and enrolled member of the WTC-HP. ii. Pre-9/11 spirometry with Forced Expiratory Volume in 1 second (FEV_1_) ≥ Lower Limit of Normal (LLN) iii. Male Firefighter status on 9/11 with exposure at the WTC-site and entry into WTC-HP before the site closure on 7/24/2002. *Exclusion Criteria*: i. lung disease prior to 9/11 as defined by positive methacholine or bronchodilator test, or FEV_1_ < LLN. ii. Not part of initial cohort in data extraction from August 1, 2017.^72^ After all inclusion/exclusion criteria applied, the baseline cohort consists of n = 4,192. *Sub-cohort Development*. A representative cohort of 20% was randomly selected (n = 837; SPSS v. 28) from the above baseline cohort, Fig. 2.

*Recruited Cohort* will be developed to assess for noninvasive biomarkers. We will recruit a subset N = 40/group (*i*. AHR only *ii*. GERD only *iii*. BE *iv*. GERD/BE and AHR overlap or v. No GERD nor AHR) from the sub-cohort, Fig. 2. Recruitment strategies will include: i. Direct mailings; ii. Email (potential participants will be sent the same IRB-approved recruitment message to their personal emails using end-to-end encryption; iii. Study website will include recruitment messages providing general information on the study and answers to frequently-asked questions. No direct communications will be made with participants through the website, and no PHI will be used or available within the study website; iv. Telephone contact. A description of the study will be provided to potential participants and, upon their expression of interest, the investigator will perform an eligibility screening. In addition to meeting the inclusion criteria as outlined above, participants should: i. have available serum from their first post 9/11 WTC-HP ii. not currently be receiving treatment for malignancy iii. have no limitations to a minimal risk blood draw iv. be willing and able to sign consent; and v. be able to attend a single-visit.

### Case Status.

#### WTC-AHR

WTC-AHR will be defined as having a positive methacholine (PC_200_ < 16), or a positive bronchodilator response (by ATS/ERS guidelines with improvement of FEV_1_ by 12% and at least 200mL) at least once post-9/11^73,74^ and/or EMR diagnosis. **GERD** will be defined as: biopsy-proven erosive esophagitis LA grade C or D; stricture or Barrett’s esophagus on endoscopy; and/or esophageal acid exposure time > 6% on a pH or pH impedance study. GERD will also be defined on EMR diagnosis and/or PPIs, H_2_ blockers, antacid, or surface agent use.^75^ **BE**, as a subset of GERD, will have any of the following additional inclusion criteria: biopsy-proven columnar epithelium lining ≥ 1cm of the distal esophagus with intestinal metaplasia characterized with goblet cells on histology; diagnosis on EMR, Table 2–3.^75^ The recruited participants will be consented prior to any research activity and measurement visit via REDCap software or in person.

### Measurement Visit.

Participant demographic information, medical history and medication history will be obtained. A physician will perform the physical examination, and verify that inclusion/exclusion criteria are met. Enrolled participants will undergo the following assessments.

### Blood Sampling.

After at least an 8 hour fast, serum and plasma will be obtained, aliquoted and banked. Each stored specimen will be assigned a unique code to ensure proper identification and linkage to the respective participant. Aliquots from the fresh samples will be assayed for complete blood count (with differential) and chemistry panel. These data are already available for the banked samples. For all samples, lipid profile, metabolomics, and protein biomarker pro ling will be performed.^10,28–30,76,77^

### Salivary Pepsin Assessment.

30mL sterile plastic tubes with 0.5 ml of 0.01M citric acid, adjusted to a pH of 2.5 (RD Biomed Ltd., Hull, UK), will be used by the participants to collect saliva in the AM (prior to brushing teeth, drinking or eating), 1h after finishing lunch, and 1h after finishing dinner.^78,79^ Participants will be instructed to cough a few times prior to spitting into the tube to clear saliva from the back of the throat and then spit into the tube. The collected samples will be stored at 4°C and analyzed within 2 days. Salivary Pepsin will be analyzed using Peptest (RD Biomed Ltd., Hull, UK) as previously described.^79^ Briely, plastic tubes will be centrifuged at 4,000 rpm for 5 minutes, and 80μL of supernatant will be added to 240μL of migration butter solution for 10 seconds. 80μL of the mixture will be added to the well of the Peptest, which contains two unique human monoclonal antibodies that detect and capture pepsin protein (specific to pepsin-3), with a lower limit of detection of 16 ng/mL and an upper limit of 500 ng/mL. A salivary pepsin level of ≥ 16 ng/mL will be considered positive. The sample will be processed in a Pepcube reader to quantify the pepsin concentration.^78^

#### Spirometry

Spirometry will be assessed using a KoKo PFT spirometer (nSpire Health Inc), and lung function assessment will be considered acceptable as per the ATS/ERS guidelines.^80^ We will select the largest acceptable measures for electronic archiving. Each participant’s predicted percentage (%) will be calculated by NHANES III equations based on their age at examination, height, sex, and race.^80,81^

#### FeNO

FeNO will be quantified using NIOX VERO^®^ (Aerocrine).^82,83^ Participants will be instructed to inhale to their total lung capacity via mouthpiece for 2–3 seconds. Then, they will exhale at a flow rate of 0.05L/second. The device will provide results in parts per billion (ppb).

#### Exhaled breath condensate (EBC)

Exhaled breath condensate (EBC) will be collected using RTubes (Respiratory Research, Inc., USA).^84^ Approximately 1–2mL of EBC sample will be obtained after 10 min of quiet normal breathing.^85^ *PH measurement*. EBC pH assay is extremely simple to perform, inexpensive, and robust, and can be easily processed on the day of collection.^86^ EBC will be de-aerated of CO_2_ by bubbling free argon gas (350ml/min) under a micro-pH reader (Orion PerpHecT micro-pH electrode) and stabilized pH will be recorded after approximately 3–5 minutes.^87^ Aliquots are then stored at −80 °C and thawed only once prior to histamine and biomarker assessment.

### Naso/oropharyngeal microbiome.

#### Collection.

Trained study team members will collect naso/oropharyngeal samples using commercially available kits (OMR-110 by DNA Genotek, Canada). Each naris will be swabbed in a circular fashion 10 times. The oropharyngeal sample will be collected by swabbing in the back of the throat in 10 circular motion to ensure sufficient swab collection. Each absorbent swab will be placed into a vial containing 1 mL of stabilizing liquid using aseptic technique. The sample will be treated with lyophilized Proteinase K, and incubated in the original vial at 50 °C for 1 hour in a water bath prior to aliquoting for long-term storage at −80 °C.

### Quality of Life, Aerodigestive Disease and End-Organ Effect Questionnaires.

#### Gastrointestinal impact

Gastrointestinal impact will be assessed using with the Patient Assessment of Upper Gastrointestinal Disorders – Quality of Life **(PAGI-QoL)** and the Patient Assessment of Upper Gastrointestinal Disorders Symptom Severity Index **(PAGI-SYM)**. Both questionnaires use a 6-point Likert scale (MAPI Research Trust).^88–91^

#### Respiratory and QoL assessment

Respiratory and QoL assessment will utilize the Health-Related Quality of Life measures **(HRQL)**^92^, St. George’s Respiratory Questionnaire **(SGRQ)**, and the Short-form-36 **(SF-36)**. HRQL assesses an individual’s perceived physical and mental health. The SGRQ is a standardized, self-administered airways disease-specific questionnaire divided into three subscales- symptoms, activity, and impact.^93^ SF-36 will capture supplemental information about their mental health, general health perception, emotional, and social role functioning.^94^

#### Cognition

Cognition will be assessed using the Montreal Cognitive Assessment (**MoCA**; version 8.1) and the Mini-Mental State Examination (**MMSE)**. MMSE is a cognitive test used to evaluate early dementia.^95,96^ Combining MoCA and MMSE can improve diagnostic utility.^97^ The MoCA will be administered by a trained/certified investigator. Members of our research team have completed MoCA training and certification through a validated MoCA cognition portal^98^ (https://mocacognition.com/). Similar to the MoCA, the MMSE assesses orientation, memory, visuospatial and language domains. Additionally, the MMSE evaluates comprehension, reading and writing.^99^ The PI will thoroughly review all scores.

### Power Analysis.

A sample size of 40 cases for each group of GERD, AHR, AHR/GERD overlap, BE, and non-GERD/non-AHR Controls (all will be subsets of AIM 1 N = 898 randomly selected cohort) achieves 80% power to detect difference as small as 0.78 SD with two-sample t-test at 0.01 significance level to account for multiple comparisons. This will allow us to achieve 80% power and significance of 0.05, based on prior studies with salivary pepsin test (personal communication with Dr. Peter Dettmar of Peptest), Fig. 2.

#### Statistical Analysis

Statistical Analysis SPSS 28 (IBM) will facilitate database management and statistics. Continuous variables expressed as mean, standard deviation (SD) if normally distributed, and as median, interquartile range (IQR) if skewed. Two-sample t-test and ANOVA will compare continuous data. Count and proportions will summarize categorical data and Pearson-χ^2^ will compare categorical data. Multivariate binary logistic regression will estimate biomarker-disease relationship for case status as a binary outcome while adjusting for confounding. Cox proportional hazards model will evaluate the effects of biomarkers, smoking, and exposure on the hazard of developing WTC-GERD or BE over time. The maximum potential effectiveness of a biomarker will be calculated by Youden Index.^100^ Goodness of t, using the Hosmer-Lemeshow test. Survival curves compared by Log-rank test. Pearson χ^2^-test will compare SABA and LABA usage between GERD, AHR, AHR/GERD overlap, BE, and non-GERD or AHR controls. Significance will be assessed by p < 0.05 for all statistical tests. Graphs will be created using Prism (v.10, GraphPad Software).

### Missing data

Variables with missing values in a small proportion of participants will be imputed using multiple imputation methods. To assess the missing at random assumption, we will evaluate the comparability between samples with missing data and those without. Sensitivity analysis will be performed by comparing the results obtained from the complete data analysis to the results obtained from multiple imputation.

### Model Building.

We have previously identified key biomarkers using a machine learning approach.^10,28–30^ We have further refined this analysis pipeline and will utilize this methodology to identify AHR, GERD, AHR/GERD overlap, and BE biomarkers. Specifically, we will utilize random forests (RF) of the ltered, normalized biomarkers. Models assessed via a modified hamming distance between variable importance rankings of models with identical hyper-parameters. A refined profile of the top 5% of important biomarkers by MDA will be included in a gradient-boosted tree model (xgboost package, R-Project) to build a classifier of AHR, GERD, AHR/GERD overlap, and BE. A random hyperparameter space search determined a nal model that maximized AUC_ROC_.

We will also use linear mixed-effects models will be used to assess the temporal trend of biomarkers with time adjusting for confounders. The longitudinal biomarkers processes will be associated to risk of developing WTC-GERD/BE using the joint modeling technique.^101^ The joint-modeling approach has become the primary method for analysis of longitudinal biomarker process and time-to-event outcome, and multiple R packages are available to implement the models. We will also consider a single index longitudinal model which enables us to reduce the dimensionality of multiple biomarkers and to evaluate joint effects of multiple biomarkers together to identify key risk factors. The single-index model incorporates longitudinal data to calculate hazard of each parameter as well as personalized dynamic risk for prognostication. Specifically, this will allow us to use a patient’s data from a single clinical exam to identify risk of GERD, AHR, overlap, or BE. Furthermore, this will allow the identification of false negatives and undertreated cases in the entire FDNY cohort.

## DISCUSSION

PM exposure, a significant component of ambient and occupational exposures is a risk factor for aerodigestive disease (such as GERD and AHR) and is associated with approximately 7-million deaths annually.^1–3,11,102–104^ GERD is the most prevalent gastrointestinal disorder in the US, with an estimate as high as 30%.^66^ Globally, the prevalence of GERD ranges from 10–25%, with an increased risk in firefighters.^52,66^ GERD is an independent risk factor in the development of BE which can lead to malignancy.^66^

Despite the similar risks, the undefirstanding of the underlying pathophysiological interrelatedness between the aerodigestive diseases (AHR, GERD and BE) remains limited. Furthermore, GERD diagnosis and treatment has been invasive and costly. Therefore, our work is focused on identifying non-invasive biomarkers which may help identify at risk populations who may benefit from earlier intervention, targeted therapies and a further undefirstanding of how their AHR is impacted by co-morbid GERD. The identification of non-invasive biomarkers of GERD/BE and the overlapping aerodigestive disease is crucial.

Our work will address the existing **knowledge gap** in aerodigestive overlap and validate biomarkers of WTC-aerodigestive disease. Biomarkers of BE may also identify individuals at risk for neoplastic disease. These findings may have broader implications for populations with GERD and PM exposure. In contrast to currently used invasive testing, noninvasive testing offers diagnostic utility with reduced risk and can direct future research into mechanisms/downstream effects. We also systematically studied biomarkers of GERD/BE and defined some of the lacunae in the non-invasive aerodigestive biomarker literature.^105^ Therefore, our Case-Control Observational Study is designed to sample a broad biomarker profile, Table 3.

### Microbiome of the Gut/Lung Axis.

Asthma susceptibility is in uenced by the gut microbiome.^106–111^ Noninvasive collection sites that can approximate the pulmonary environment are of key interest. Studies have failed to show that the microbiomes of induced sputum were similar to the lung.^112,113^ Noninvasively collected oropharyngeal and nasopharyngeal swabs in conjunction could approximate the lung microbiome.^114^ Research has revealed that the esophageal microbiome undergoes alteration in individuals with GERD, BE, and other motility disorders.^115,116^ Although these findings highlight the potential role of the microbiome studies in the diagnosis and therapeutic approaches for aerodigestive disease, further studies are needed and will be one of the key readouts planned in our study.

#### EBC

EBC analysis holds great promise in addressing unmet medical needs by expanding the portfolio of noninvasive assays for the multiple coexisting pathological mechanisms underlying respiratory disorders and GERD. Compounds identified in EBC include histamine, adenosine, ammonia, hydrogen peroxide, isoprostanes, leukotrienes, nitrogen oxides (NOx), peptides, cytokines, protons and various ions.^85^ Histamine plays a vital role in digestion but elevated levels can contribute to the development of GERD.^117,118^

#### Salivary pepsin

Salivary pepsin has been studied in several GERD biomarker studies.^105^ Due to the overlap of various Refux symptoms with other GI pathologies, the diagnosis of GERD can be challenging. However, salivary pepsin test offers a simple and convenient way for detecting Refux through salivary sample collection, providing quick and non-invasive results. Compared to other diagnostic modalities, this approach is time-efficient and requires much less effort.^119^ Moreover, pepsin measurements can identify pathologic Refux even in the absence of symptoms, and remain unaffected by the concurrent use of PPI. Several studies have demonstrated that pepsin detection in the sputum and/or saliva can be regarded as a sensitive, non-invasive method for the diagnosis of the proximal Refux of gastric contents, with a sensitivity ranged from 41.5–73% and high specificity of 86.2 to 98.2%.^78,79^ Despite these findings little is known about pepsin in the context of aerodigestive co-morbid disease.

#### FeNO

FeNO, a biomarker of lung disease activity, will be a valuable measure in our population. FeNO is associated with airway hyperreactivity, and several studies demonstrated that FeNO is increased during obstructive exacerbations.^120^ In our population with the aerodigestive overlap, FeNO levels can serve as an indicator of potential underlying AHR exacerbating symptoms of GERD. Thus, our work will also contribute to understand the role of FeNO in GERD, which remains inconclusive as only a limited number of studies have examined AHR/GERD.^121,122^ The detrimental impact of even once-weekly episodes of GERD on quality of life^123^ highlighted the importance of assessing aerodigestive disease quality of life and disease activity, therefore we will quantify the effects of GERD on these aspects through a validated set of questionnaires that will assess QoL, GERD specific symptoms and also cognitive involvement.

Non-invasive biomarkers of GERD, BE, AHR, treatment efficacy, and severity of symptoms will also be assessed in serum. This will allow us to measure Tumor Necrosis Factor (TNF-α), C-peptide, Fractalkine and Interferon-gamma-induced Protein 10 (IP-10) in our case cohort study to validate our prior pilot study.^1–3^ Serum samples will also be used to perform metabolomic pro ling that will allow us to investigate metabolic correlates of aerodigestive disease. In addition, by validating serum biomarkers (proteins and metabolome) of GERD/BE, we seek to provide a biologically plausible target that enables early detection and facilitates therapeutic intervention in the PM exposed populations. Moreover, non-invasive phenotyping of WTC aerodigestive disease holds promise in improving the sensitivity and specificity of GERD diagnosis, enabling earlier identification of BE and facilitate the development of personalized therapy, thus to improve both the quality of life and overall health outcomes.

### Limitations and potential study concerns.

We envision there are several limitations of our study. It is possible that no significant association exists between noninvasive biomarkers and aerodigestive diseases in the second decade after WTC exposure. The generalizability of our study could be impacted because the FDNY source cohort had no aerodigestive disease prior to 9/11 and had their serum samples banked within six months of 9/11, therefore making it less comparable to other cohorts. There may also be a subset of patients without history of GERD, but could still receive a clinical diagnosis of GERD based on questionnaires and/or elevated pepsin/biomarkers. For these patients, further follow-up with a gastroenterologist will be recommended. Additionally, we may use FeNO levels to identify the potential underlying AHR exacerbating symptoms associated with GERD. We will also account for the potential risk of loss to follow-up regarding the completion of the questionnaires and attendance of the in-person visit.

Further investigation into the overlap of GERD/BE and AHR is envisioned to provide valuable insights in distinguishing disease phenotypes, demonstrating that biomarkers can predict GERD and/or BE. This work will have clinical implications for the diagnosis and treatment of WTC associated disease, as well as for the management of other patients in the WTC monitoring programs, and for the general population as intense PM exposures are occurring more frequently, for example through wild fire related PM. Our research will contribute to the development of a robust biomarker set with optimal explanatory power when applied to diverse cohorts.

## Data Availability

The datasets used and/or analyzed during the current study are available from the corresponding author on reasonable request.

## Abbreviations

AHR: Airway Hyper reactivity
BADBURN: Biomarkers of Airway Disease, Barrett’s and Underdiagnosed Refux **N**oninvasively
BMI: Body Mass Index (kg/m^2^)
COPD: Chronic Obstructive Pulmonary Disease
DBP: Diastolic Blood Pressure
DSMB: Data and Safety Monitoring Board
EAC: esophageal adenocarcinomas
ECG: Electrocardiogram
FDNY: Fire Department of New York
FE_NO_: Fractional Exhaled Nitric Oxide
FEV_1_: Forced Expiratory Volume in 1 second
FVC: Forced Vital Capacity
HRQL: Health-Related Quality of Life
HP: Health Program
IRB: Internal Review Board
LI: lung Injury
LLN: Lower Limit of Normal
LoCalMed: Low Calorie Mediterranean Diet
MetSyn: Metabolic Syndrome
MoCA: Montreal Cognitive Assessment
MMSE: Mini-Mental State Examination
MOP: Manual of Procedures
MND: MyNetDiary
N: Number
NIH: National Institutes of Health
NYU: New York University
OAD: Obstructive Airways Disease
PAGI-QOL: Patient Assessment of Upper Gastrointestinal Disorders-Quality of Life
PAGI-SYM: Patient Assessment of Upper Gastrointestinal Disorders-Symptoms Severity
PI: Principal Investigator
PM: Particulate Matter
PWV: Pulse Wave Velocity
QoL: Quality of Life
RCT: Randomized Clinical Trial
SBP: Systolic Blood Pressure
SF-36: Short-Form 36
SGRQ: St. George’s Respiratory Questionnaire
SOP: Standard Operating Procedure
US: United States
WTC: World Trade Center
9/11: September 11, 2001
